# Interannual Dynamics of Macrobenthic Communities near a Coastal Nuclear Power Plant: Environmental Drivers and Risks of Cooling Source Blockage

**DOI:** 10.3390/biology15110890

**Published:** 2026-06-04

**Authors:** Wen Huang, Wenbin Zhang, Wei Liu, Lijing Fan, Dong Wen, Biqi Zheng, Zefeng Yu, Shouwei Yu

**Affiliations:** 1 Key Laboratory of Marine Ecological Monitoring and Restoration Technology, Ministry of Natural Resources, Shanghai 201206, China; 2 Ningde Marine Center, Ministry of Natural Resources, Ningde 352100, China; 3 Joint Research Center of Marine Ecology of Coastal NPP, Ningde 352100, China; 4 Fujian Ningde Nuclear Power Co., Ltd., Ningde 355200, China

**Keywords:** cooling water systems, macrobenthos, community structure, GMRI, potential risk organisms

## Abstract

Cooling water systems of coastal nuclear power plants in China are frequently threatened by blockages caused by marine organisms. However, long-term studies on macrobenthic community dynamics and their associations with environmental factors are scarce, limiting the precise prevention of such blockage risks. This study conducted quantitative monitoring of macrobenthos and synchronous measurement of water environmental factors at 24 sampling stations in three functional areas (water intake, harbor basin, and drainage outlet) adjacent to the Northeast Fujian NPP from 2018 to 2024. Community structure characteristics were analyzed using the Shannon–Wiener and Margalef indices. The Grappler Method Risk Index (GMRI) was employed to screen species at risk of blocking cooling water systems, and the Mantel test and random forest models were applied to explore the associations between the macrobenthic community and environmental factors. A total of 161 macrobenthic species were identified. Polychaetes (71 species, accounting for 44.1%) were the absolute dominant group, followed by crustaceans (35 species) and Mollusks (30 species). The interannual fluctuation range of the polychaete proportion was 41.1–57.8%, reaching a peak in 2023. There were significant differences in community structure among different areas (PERMANOVA, *p* < 0.05), with the largest inter-regional difference in 2024 (R^2^ = 0.36). The annual average number of species (9 species), density (155.25 ind./m^2^), and biomass (29.58 g/m^2^) in the drainage outlet were higher than those in the water intake and harbor basin. The GMRI identified *Protankyra bidentata* (spiny sea cucumber, GMRI values of 50.67% to 64.98% from 2019 to 2023) and *Actiniaria* sp. (sea anemone, a GMRI value of 54.63% in 2021) as medium-risk species for cooling water system blockage, while most other organisms were classified as low risk or extremely low risk. The Mantel test and random forest analysis confirmed that nitrogen nutrients (NO_3_^−^) and phosphorus (PO_4_^3−^) were significantly positively correlated with the polychaete community. Furthermore, NO_3_^−^ and NH_4_^+^ each explained 13.66% of the variation in the diversity index (*H*′), serving as key factors driving community structure. This study demonstrates the co-dominance of thermal and nutrient drivers in shaping macrobenthic communities over a multi-year scale, and identifies specific, morphologically suited taxa as potential blockage risks. The findings provide a scientific basis for targeted risk-species monitoring and support the integration of long-term ecological data into NPP cooling water system security management.

## Simple Summary

In recent years, blockages caused by marine organisms have increasingly threatened the cooling water systems of coastal nuclear power plants (NPPs). This study investigated the macrobenthic communities in three functional areas (water intake, harbor basin, and drainage outlet) adjacent to the Northeast Fujian NPP from 2018 to 2024. We analyzed community structure (species composition, biomass, density, and diversity) and its correlation with environmental factors. The results showed that the environmental factors affecting the changes in the macrobenthic community structure varied across different years in the waters adjacent to the Northeast Fujian NPP. Using the Grappler Method Risk Index, only two species (*Protankyra bidentata* (spiny sea cucumber) and *Actiniaria* sp. (sea anemone)) were identified as medium risks for blocking cooling water systems, while most other organisms were classified as low risk or extremely low risk. The Mantel test and random forest analysis revealed that nitrogen nutrients (NO_3_^−^) and phosphorus (PO_4_^3−^) were significantly positively correlated with the polychaete community. Additionally, NO_3_^−^ and NH_4_^+^ each explained 13.66% of the variation in the diversity index (*H*′), serving as key factors driving the macrobenthic community structure. This study provides scientific support for establishing a marine biological risk early-warning system and for the ecological security management of coastal NPPs.

## 1. Introduction

The abnormal aggregation (e.g., outbreaks and biofouling) of marine organisms poses a significant threat to the safety of water intake in cooling water systems in coastal nuclear power plants (NPPs) [1,2,3]. Over the past few decades, such phenomena have compromised the safety of cooling water systems in coastal nuclear power plants worldwide [4,5,6,7]. For instance, in December 2009, Unit 4 of France’s CRUAS Nuclear Power Plant experienced a cooling source failure due to waterweed blockage [8]. Swedish nuclear power plants were forced to shut down following jellyfish outbreaks [9]. South Korea’s Uljin Nuclear Power Plant experienced a 38% drop in power generation capacity due to blockage by *Salpa fusiformis* [10]. In China, similar challenges have been reported, such as the shutdown of the Hongyanhe NPP following an *Aurelia aurita* bloom [11] and reduced filter efficiency at the Ningde NPP due to *Acaudina molpadioides* [12,13]. Such incidents have resulted in economic losses totaling billions of US dollars [2,14]. According to statistics, among nearly 200 nuclear power plant water intake blockage incidents globally, 84% were triggered by marine organisms [4,15]. Therefore, systematic research on the mechanisms behind marine organism-induced blockages is urgently needed to inform effective prevention strategies.

Previous studies have mainly focused on marine organism outbreaks [3,4,16,17], acoustic imaging and numerical simulation [16,18,19,20], optimization of water intake design [21,22], accumulation of marine debris [23], and early warning and prevention/control methods [2]. Meanwhile, monitoring and early warning systems, as well as decision support systems targeting marine organisms in NPP cooling water systems, have been developed and put into use [8,24,25,26,27,28,29]. Some scholars have predicted biomass in waters adjacent to NPPs by establishing models [30,31], identifying the area within 1 km of the cooling source as a high-risk zone. Others have attempted to integrate acoustic imaging with numerical simulation, using multi-beam sonar [16] or the FVCOM model [32] to predict the migration pathways of risk-associated organisms.

However, existing research on marine organism-induced blockage risks in NPP cooling water systems still has shortcomings. Wu et al. (2023) [17] found that the abundance of spring zooplankton in Daya Bay reached as high as 1372.01 ind./m^3^, and aggregation of gelatinous organisms (*Dolioletta gegenbauri*) is prone to causing blockages; however, their study was based only on one year of survey data. He et al. [33] and Wang Yu et al. [7] screened multiple high-risk phytoplankton species that tend to form communities or aggregations by combining one year of survey data with historical data. Hu et al. [34] analyzed the thermal discharge effects of China’s Daya Bay NPP and found that a 5–10 °C increase in surface water temperature significantly alters phytoplankton community composition, though this finding was based on a single survey and limits its reflection of temporal dynamics. Huang et al. (2025) [5] conducted an annual survey of nekton in waters adjacent to Northeast Fujian NPP, indicating that the risk of nekton induced cooling water blockage is highest between May and October. Lou et al. (2023) [35] simulated the movement of krill using 250,000 passive particles and found that *Acetes chinensis* forms “path-dependent aggregation” through the Lagrangian Flow Network (LFN) after 30 days of tracking. Most of the aforementioned studies are based on short-term survey data (one year or less), making it difficult to reflect the long-term dynamics of the macrobenthic community structure. Simultaneously, research on the correlations between macrobenthic communities and environmental factors remains insufficient.

Although Northeast Fujian NPP has conducted research on macrobenthos [12,13,36,37], jellyfish [38,39], plankton [40,41], and nekton [5,42], a comprehensive understanding of long term time series monitoring of macrobenthos is still lacking, especially the changing trends of the biological community structure on a nearly 5-year time scale and their long-term response mechanisms to environmental factors. Therefore, based on nearly 5 years of survey data (2018–2024) from the benthos of the water intake, harbor basin, and drainage outlet of Northeast Fujian NPP, this study analyzes the interannual changes in species composition, density, biomass, diversity indices, and dominant species of macrobenthos in this area. It also screens and evaluates macrobenthos species that may block the cooling water system, identifies potential risk-associated species, and explores the correlations between community structure and environmental factors. This research provides scientific data to support early warnings of macrobenthos risk, ecological security management, and cooling water system protection of coastal NPP projects.

## 2. Materials and Methods

### 2.1. Sample Stations

**Figure 1:**
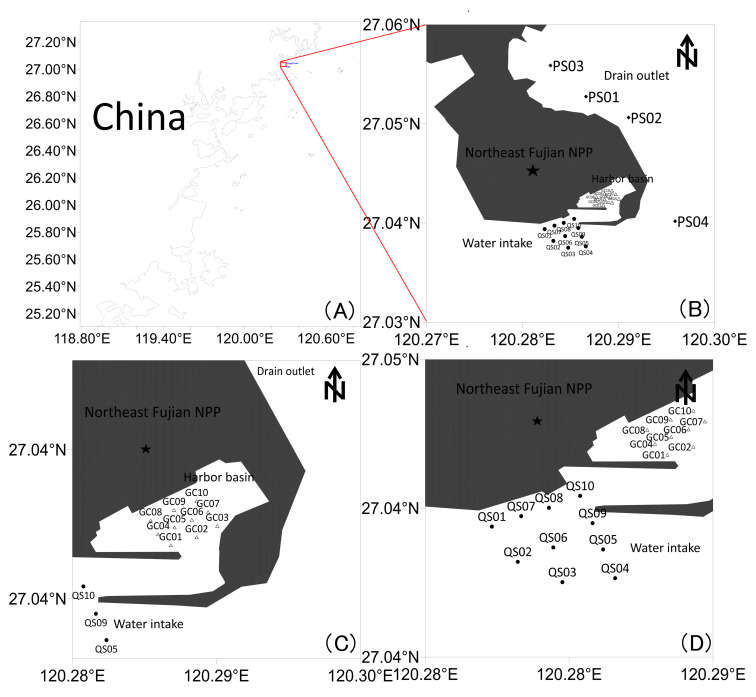
Map of sampling stations adjacent to the Northeast Fujian Nuclear Power Plant. (**A**) Geographic overview map. (**B**) Detailed map of the drain outlet zone (PS01–PS04). (**C**) Detailed map of the harbor basin zone (GC01–GC10). (**D**) Detailed map of the water intake zone (QS01–QS10). Star represents the location of Northeast Fujian Nuclear Power Plant.

This study was conducted in the waters adjacent to the Northeast Fujian Nuclear Power Plant (NPP), Fujian, China (27.03° N–27.06° N, 120.27° E–120.30° E). The data used in this study were derived from the quantitative monitoring of macrobenthos carried out by Ningde Marine Center in the study area from 2018 to 2024. Sampling was not conducted in 2022 due to safety concerns from frequent typhoons. A total of 24 sampling stations were deployed, with sampling conducted annually in either July or October. Based on the hydrological characteristics of the NPP-adjacent waters, the monitored area was divided into three functional zones, including water intake (QS01–QS10), drainage outlet (PS01–PS04), and harbor basin (GC01–GC10), with 10, 4, and 10 sampling stations, respectively (Figure 1).

### 2.2. Macrobenthos Collection and Analysis

Biological samples were collected in accordance with the protocols specified in the Marine Monitoring Specifications [43]. A 0.1 m^2^ grab sampler was employed, and two replicates were collected at each station and combined into one quantitative sample. Sediment samples were rinsed through a sieve with an aperture of 0.5 mm to retain macrobenthos. Biomass was weighed using an electronic balance with a precision of 0.001 g (Model WT3003, Serial Number 1999). The taxonomy of macrobenthic species followed the World Register of Marine Species (WoRMS) database [44] (http://www.marinespecies.org, accessed on 25 April 2025) and was cross-verified against *Checklist of Marine Biota of China Seas* [45].

### 2.3. Environmental Factor Measurement

Concurrent with biological sampling, key water quality parameters were monitored at each station following the national standard *Marine Monitoring Specifications—Part 4: Seawater Analysis* (GB 17378.4-2007) [46]. Water quality parameters included temperature (WT, °C), salinity (SAL), pH, dissolved oxygen (DO, mg/L), chemical oxygen demand (COD, mg/L), reactive phosphate (PO_4_^3−^, mg/L), nitrite (NO_2_^−^, mg/L), nitrate (NO_3_^−^, mg/L), ammonium (NH_4_^+^, mg/L), and suspended solids (SS, mg/L).

### 2.4. Data Analysis

The interannual changes in the community structure of macrobenthos in the waters adjacent to Northeast Fujian NPP were analyzed using the Shannon–Wiener index (*H*′) [47], Margalef’s richness index (*d*) [48], and Pielou’s evenness index (*J*′) [49], with calculation methods referring to the cited studies.

Dominance (Y) was used to identify dominant species at each station. A species was considered dominant when $Y_{i}$ > 0.02 [50], calculated as follows:

(1) $$Y_{i}\;=\;P_{i}\;\times \;f_{i}$$

where $f_{i}$ = the frequency of occurrence of the i-th species across all sampling stations.

The risk of macrobenthos causing cooling water system blockage was evaluated using the Grappler Method Risk Index (GMRI) [12], which was developed by Cai et al. (2022) specifically for assessing the risk of benthic macrofauna entrapment in coastal nuclear power plant cooling water systems.

GMRI = (A + B + P + F + C) × 20%(2)

A (percentage of abundance) = the percentage of the average density of the target species relative to the average density of all macrobenthos.

B (percentage of biomass) = the percentage of the average biomass of the target species relative to the average biomass of all macrobenthos; P (percentage of sampling stations) = the frequency of occurrence of the target species across all stations; F (floating risk) = the potential of the species to float with water flow; and C (water-flow-driven risk) = the potential of the species to be transported by water flow to the cooling water intake.

Risk classification based on GMRI values: GMRI ≥ 90%, extremely high risk; 70% ≤ GMRI < 90%, high risk; 50% ≤ GMRI < 70%, medium risk; 30% ≤ GMRI < 50%, low risk; and GMRI <30%, extremely low risk.

### 2.5. Statistical Analysis

SPSS 27 software was used to perform one-way Analysis of Variance (ANOVA) on environmental factors, with the water intake, harbor basin, and drainage outlet as fixed factors. Prior to analysis, the Shapiro–Wilk test was used to verify data normality, and the Levene test was used to check homogeneity of variances. Water quality environmental factors were subjected to logarithmic transformation (log_10_(raw data + 1)) to meet the requirements of normality and homogeneity of variances. For data with homogeneous variances, Tukey’s HSD test was used for post hoc multiple comparisons.

R software (version 4.10) was used for subsequent analyses. Species abundance data were subjected to a square root transformation, and Principal Coordinates Analysis (PCoA) was constructed based on the Bray–Curtis similarity matrix. Permutational Multivariate Analysis of Variance (PERMANOVA) with 999 permutations was used to test inter-group differences.

The Mantel test (linkET package version 0.0.7.4) was performed to analyze the correlation between macrobenthic community structure and environmental factors.

Random forest analysis (randomForest package version 4.7.1.1) was conducted with the diversity index (*H*′) as the response variable and environmental factors as explanatory variables. Factor importance was calculated using node impurity (Gini coefficient) with 1000 permutations.

Graphs of species composition and diversity trends were plotted using Prism 10.0. One-way ANOVA combined with Tukey’s post hoc test was used to compare differences in biological indices across different areas, with the significance level set at *p* < 0.05. The sampling station map was prepared using Surfer 16 software (https://www.goldensoftware.com/products/surfer/ (accessed on 25 November 2025)).

## 3. Results

### 3.1. Species Composition of Macrobenthos

**Figure 2:**
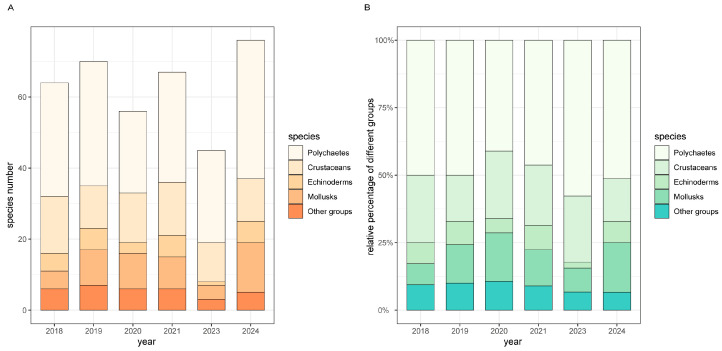
Interannual variation in number of species of macrobenthic taxa from 2018 to 2024. (**A**) Total species number of different macrobenthic taxa. (**B**) Relative percentage of different macrobenthic taxa.

From 2018 to 2024, quantitative monitoring samples from the waters adjacent to Northeast Fujian NPP were analyzed, resulting in the identification of 161 macrobenthic species, among which 12 were identified at the genus level. Polychaetes were the most abundant group, with 71 species accounting for 44.1% of the total, followed by crustaceans (35 species, 21.7%), Mollusks (30 species, 18.6%), and Echinoderms (10 species, 6.2%). Other groups included Chordates, Cnidarians, Latyhelminths, Nemerteans, Sipunculans, and Echiurans (Table 1, Figure 2).

Polychaetes remained the dominant group, accounting for 50.0% of the total species in 2018 and 2019, decreasing to 41.1% in 2020, and then recovering to 46.3% in 2021 and 51.3% in 2024, with a peak of 57.8% in 2023 (with an interannual fluctuation range from 41.1% to 57.8%). Crustaceans, the second-largest group, decreased year by year from 25.0% in 2018 to 15.8% in 2024, with a slight recovery to 25.0% in 2020 (interannual fluctuation range from 15.8% to 25.0%). Mollusks, the third-largest group, increased significantly to 14.3% in 2019 and 17.9% in 2020, dropped to 8.9% in 2023, and rose again to 18.4% in 2024 (interannual fluctuation range from 7.8% to 18.4%). Echinoderms, the fourth-largest group, accounted for 8.6% in 2019, decreased to 5.4% in 2020, recovered to 9.0% in 2021, fell to 2.2% in 2023, and rebounded to 7.9% in 2024. In most years, only one species was recorded in other groups (Cnidarians, Platyhelminths, Nemerteans, Sipunculans, and Echiurans).

**Figure 3:**
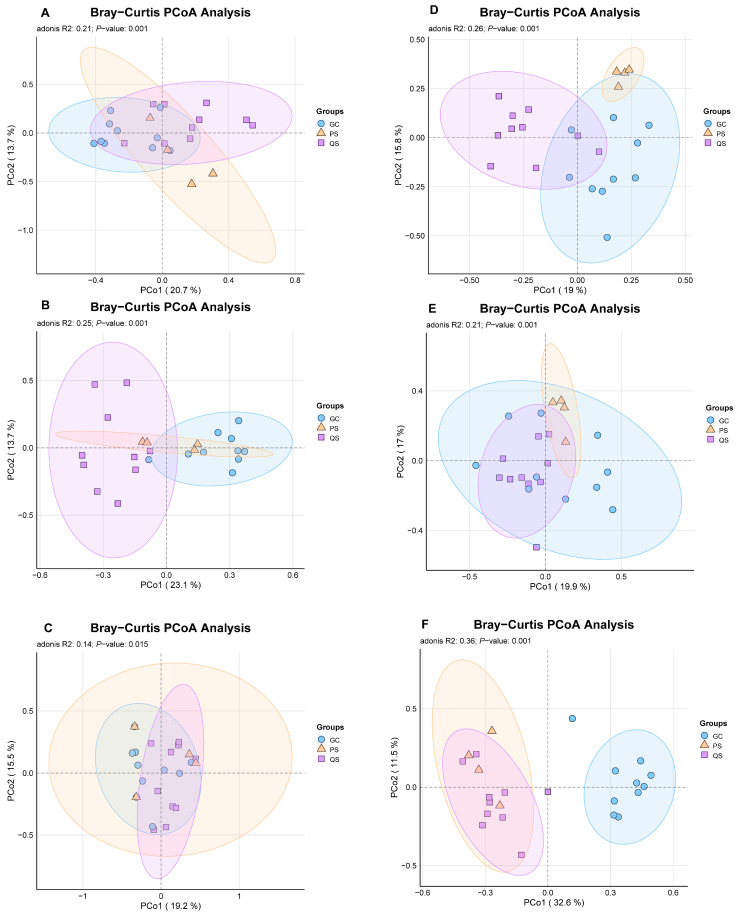
PCoA plots of macrobenthic communities with PERMANOVA test from 2018 to 2024. Different colors represent functional zones: purple = water intake (QS); blue = harbor basin (GC); yellow = drainage outlet (PS). (**A**) 2018 PCoA analysis of macrobenthic communities; (**B**) 2019 PCoA analysis of macrobenthic communities; (**C**) 2020 PCoA analysis of macrobenthic communities; (**D**) 2021 PCoA analysis of macrobenthic communities; (**E**) 2023 PCoA analysis of macrobenthic communities; (**F**) 2024 PCoA analysis of macrobenthic communities.

The results of Principal Coordinates Analysis (PCoA) and Permutational Multivariate Analysis of Variance (PERMANOVA) (Figure 3) showed significant differences in macrobenthic communities among different areas. In 2018, PC1 accounted for 20.7% of the total variance with clear significant differences among groups (Figure 3A). In 2019, PC1 accounted for 23.1%, reflecting more pronounced inter-group differences than those observed in 2018 (Figure 3B). In 2020, although statistical significance was slightly attenuated, distinct differences still persisted among groups with PC1 and PC2, explaining 19.2% and 15.5% of the variance, respectively (Figure 3C). The year 2021 saw PC1 contributing 19% and PC2 contributing 15.8% of the total variance (Figure 3D). In 2023, PC1 and PC2 accounted for 19.9% and 17% of the variance, respectively, with significant inter-group differences remaining evident (Figure 3E). Notably, 2024 showed the most striking pattern across all study years as PC1 alone explained 32.6% of the variance, indicating that inter-group differences were particularly prominent along the PC1 axis and sample separation was consequently more distinct (Figure 3F). Overall, there were significant differences in macrobenthic community structure among groups in each year, with the highest inter-group difference in 2024 and the relatively lowest in 2020.

### 3.2. Number of Species, Biomass, Density, and Diversity Indices of Macrobenthos

From 2018 to 2024, the annual number of macrobenthic species in the study area ranged from 45 to 76, with an annual average of 63. The total number of species showed a fluctuating upward trend, reaching the highest point in 2024 (an increase of approximately 20% compared to 2018). At the group level, the number of species in major groups such as polychaetes and crustaceans also showed a fluctuating upward trend, while those of Mollusks and Echinoderms remained relatively stable, and the number of species in other groups exhibited a slight downward trend.

In terms of spatial distribution, the number of species in the water intake (QS), harbor basin (GC), and drainage outlet (PS) was generally low and showed a fluctuating pattern. One-way ANOVA revealed no significant differences in the number of macrobenthic species across the three areas in 2019, 2020, 2021, and 2023 (*p* > 0.05), while significant differences were observed in 2018 (F_2_ = 5.14, *p* < 0.05), and highly significant differences were observed in 2024 (F_2_ = 11.62, *p* < 0.01).

From 2018 to 2024, the annual average density of macrobenthos in the study area ranged from 38.26 ind./m^2^ to 220.29 ind./m^2^, with an annual mean of 97.33 ind./m^2^, reaching the highest in 2024 and the lowest in 2020. The annual biomass ranged from 7.76 g/m^2^ to 24.01 g/m^2^, with an annual mean of 16.98 g/m^2^, peaking in 2020 and bottoming out in 2023.

Spatial differences were observed among the three areas with the drainage outlet (PS) exhibiting the highest annual average number of species (9), density (155.25 ind./m^2^) and biomass (29.58 g/m^2^) in comparison to the harbor basin (GC) which had 7.3 species, density (80.86 ind./m^2^) and biomass (15.09 g/m^2^) and the water intake (QS) which had nine species, density (110.50 ind./m^2^) and biomass (14.49 g/m^2^). Across all sampling years, the drainage outlet (PS) recorded the highest number of species density and biomass in 2024.

One-way ANOVA showed no significant differences in macrobenthic density among the three areas in 2019, 2020, 2021, and 2024 (*p* > 0.05), but significant differences were detected in 2018 (F_2_ = 6.01, *p* < 0.01) and 2023 (F_2_ = 4.466, *p* < 0.05). For biomass, no significant differences were found among the three areas in 2018, 2019, 2021, 2023, and 2024 (*p* > 0.05), while highly significant differences were observed in 2020 (F_2_ = 6.713, *p* < 0.01).

The macrobenthic community diversity index (H′) in the waters adjacent to Northeast Fujian NPP ranged from 2.15 to 2.93, with an annual average of 2.57, reaching the highest value in 2024. The richness index (d) ranged from 0.80 to 1.28 (annual average value 1.09), and the evenness index (J′) ranged from 0.79 to 0.94 (annual average value 0.90).

No significant differences were found in the diversity index (*H*′) among areas in 2019, 2020, 2021, and 2023 (*p* > 0.05), but significant differences were observed in 2018 (F_2_ = 4.30, *p* < 0.05) and highly significant differences were identified in 2024 (F_2_ = 16.11, *p* < 0.01). No significant differences in the richness index (d) were detected among areas in 2019, 2020, 2021, and 2023 (*p* > 0.05), while significant differences were found in 2018 (F_2_ = 4.25, *p* < 0.05) and highly significant differences in 2024 (F_2_ = 18.12, *p* < 0.01). No significant differences in the evenness index (J′) were observed among areas in 2018, 2019, 2020, 2021, and 2024 (*p* > 0.05), but significant differences were found in 2023 (F_2_ = 5.48, *p* < 0.05).

Overall, no obvious consistent differences were observed in the diversity, richness, and evenness indices among the water intake, harbor basin, and drainage outlet. Comprehensively considering the three indices, the biodiversity in the drainage outlet was lower than that in other areas in most years.

### 3.3. Dominant Species, GMRI, and Their Correlations with Environmental Factors

The dominant species of macrobenthos and their corresponding dominance values in the waters adjacent to the NPP during the period 2018–2024 (Table 2). During this period, the primary dominant species were *Notomastus* cf. *aberans*, *Protankyra bidentata*, and *Heteromastus filiformis*. In 2018, the dominant species at the water intake (QS) was *Gammaridean* sp. with a dominance value of 0.148, while the dominant species at the harbor basin (GC) was *Notomastus* cf. *aberans* (0.072) and that at the drainage outlet (PS) was *Notomastus* cf. *aberans* (0.079). In 2019, the dominant species at QS was *Xantho distinguendus* (0.097), while that at GC was *Protankyra bidentata* (0.185) and that at PS was *Notomastus* cf. *aberans* (0.125). In 2020, the dominant species at QS was *Aglaophamus dibranchis* (0.066), while that at GC was *Nassarius semiplicatus* (0.045) and that at PS was *Notomastus* cf. *aberans* (0.129). In 2021, the dominant species at QS was *Actiniaria* sp. (0.190), whereas that at GC was *Protankyra bidentata* (0.430) and that at PS was *Paraprionospio* sp. (0.040). In 2023, *Heteromastus filiformis* was the dominant species at both QS (0.165) and PS (0.667), while that at GC was *Notomastus* cf. *aberans* (0.151). In 2024, the dominant species at QS was *Gammaridean* sp. (0.355), whereas that at GC was *Polydora* sp. (0.694) and that at PS was *Sternaspis scutata* (0.082). Among these dominant taxa, polychaetes were predominantly represented by *Notomastus* cf. *aberans*, Echinoderms were represented by *Protankyra bidentata*, and crustaceans were represented by *Xantho distinguendus* and *Gammaridean* sp. A total of 19 dominant species were recorded at QS, with *Xantho distinguendus*, *Glycera chirori*, and *Gammaridean* sp. occurring in more than two years.

At GC, 23 dominant species were identified, with *Notomastus* cf. *aberans* and *Protankyra bidentata* species occurring in more than two years, and *Notomastus* cf. *aberans* species appearing continuously dominant in 2018, 2021, and 2023. At PS, 15 dominant species were documented, with *Notomastus* cf. *aberans* persistently dominant from 2018 to 2023. Overall, *Notomastus* cf. *aberans* and *Protankyra bidentata* were distributed as dominant species across all three areas, with *Heteromastus filiformis* also found to be a common dominant taxon. *Protankyra bidentata* predominantly inhabits muddy substrates of coastal shallow seas and is widely distributed from the Bohai Sea to the Beibu Gulf of China; it is a prevalent species in nearshore ecosystems. *Actiniaria* sp. prefers hard substrates near water intakes, where it attaches to rocks or artificial structures and secretes viscous substances. Other dominant species, such as *Notomastus* cf. *aberans* and *Heteromastus filiformis*, are typical detritivores adapted to high-nutrient sediment environments.

Based on the density, biomass, and collection frequency of macrobenthos obtained in the waters adjacent to Northeast Fujian NPP from 2018 to 2024, the GMRI was calculated for 11, 12, 10, 15, 10, and 13 macrobenthic species in each respective year (Table 3). The results indicated that *Protankyra bidentata* (spiny sea cucumber) and *Actiniaria* sp. (sea anemone) posed a medium risk of blocking the NPP’s cooling water system, while most other organisms were classified as low risk, and some others as extremely low risk.

The highest GMRI value (64.98%) was recorded for *Protankyra bidentata* in 2019, and the lowest (18.62%) value was recorded for *Trigonothracia jinxingae* in 2024. For *Protankyra bidentata*, the GMRI values reached 64.98% (July 2019), 50.67% (October 2020), 60.80% (July 2021), and 52.51% (October 2023), all falling into the medium-risk category. Additionally, its biomass proportion ranged from 11.75% to 53.66% during these periods. The GMRI of *Actiniaria* sp. only reached 54.63% (medium risk) in July 2021.

**Figure 4:**
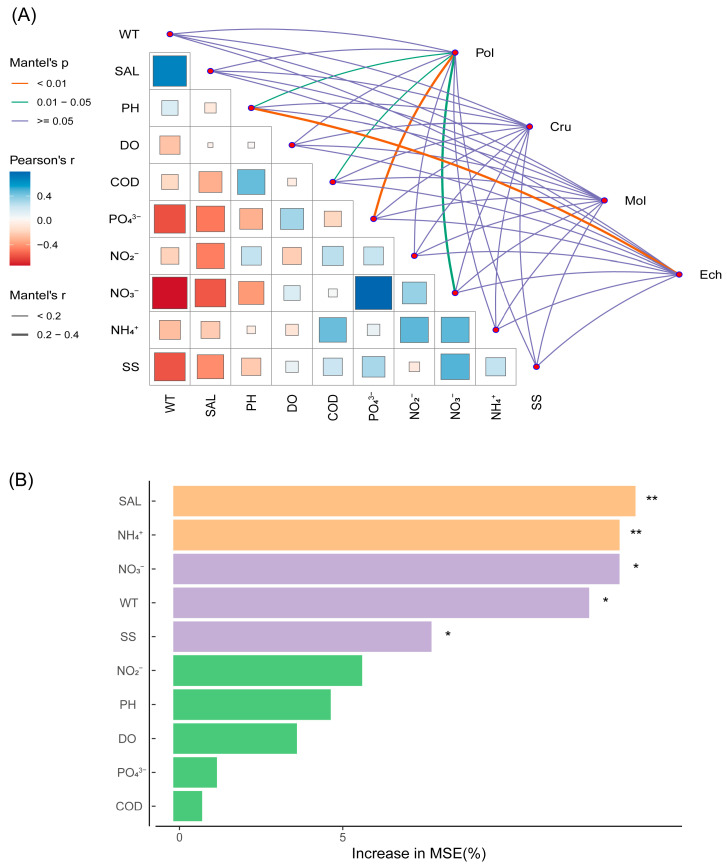
(**A**) Macrobenthic Mantel analysis; (**B**) key environmental factors affecting *H*′ identified by random forest analysis. * indicates significant importance at *p* < 0.05, and ** indicates highly significant importance at *p* < 0.01.

The results of the Mantel test analysis between subtidal macrobenthic communities and environmental factors from 2018 to 2024 showed that waterborne PO_4_^3^^−^ had a highly significant effect on the polychaete community (*p* < 0.01), and the community was also significantly affected by pH, COD, and NO_3_^−^ (*p* < 0.05). For the echinoderm community, water pH exerted a highly significant effect (*p* < 0.01) (Figure 4A).

Random forest analysis of the correlation between the macrobenthic diversity index (*H*′) and environmental parameters indicated that the key water quality factors influencing *H*′ were SAL (Salinity), NH_4_^+^, NO_3_^−^, WT (water temperature), and SS (suspended solids). These factors explained 14.14%, 13.66%, 13.66%, 12.73%, and 7.90% of the variation in *H*′, respectively (Figure 4B).

## 4. Discussion

### 4.1. Spatio-Temporal Dynamics of the Macrobenthic Communities

The results of this study revealed significant interannual variation and spatial differences in the macrobenthic community structure adjacent to Northeast Fujian NPP. Throughout the monitoring period, polychaetes remained the dominant taxonomic group. This persistent dominance is closely linked to their high physiological tolerance to environmental perturbations. Most polychaetes possess euryhaline and eurythermal traits [51], enabling them to withstand moderate thermal fluctuations and salinity variations associated with coastal runoff and NPP thermal discharge. Their capacity for cutaneous respiration also permits survival in episodically hypoxic conditions, particularly near the drain outlet (PS), where organic matter accumulates.

Thermal discharge from the Northeast Fujian NPP increases local seawater temperatures by 2.0–3.0 °C relative to the intake (QS) and harbor basin (GC) areas (Table 4). This warming likely accelerates organic matter decomposition in the water column and sediments [52], increasing the availability of detrital resources for deposit-feeding polychaetes. The enhanced food supply probably supports their numerical dominance and sustained population growth over the years. Spatially, biomass and density were consistently highest in the PS zone across most years. This pattern aligns with the thermal enhancement of metabolic rates and organic matter turnover, which collectively create a more favorable habitat for polychaetes [23,53] and is consistent with previous studies reporting elevated macrobenthic abundance near thermal discharge plumes [54,55]. Thermal input may also weaken water column stratification, promote nutrient upwelling, and modify microhabitat conditions in ways that further facilitate macrobenthic growth [23].

However, one-way ANOVA indicated no statistically significant spatial differences in univariate community metrics among the three zones in most years. This suggests that the operational impacts of the NPP remain within the coastal ecosystem’s current capacity for self-regulation. Nevertheless, the long-term cumulative effects of thermal discharge and nutrient enrichment on community structure warrant continued monitoring, as subtle shifts in species composition and functional traits may emerge over time.

In contrast to polychaetes, the relative abundance of crustaceans displayed a clear declining trend. This decrease is likely associated with sediment hypoxia driven by eutrophication, which can impair the respiratory physiology of crustaceans—a group generally sensitive to low dissolved oxygen (DO) levels. The PS area, in particular, is prone to seasonal DO depletion due to high organic loading, which may suppress reproduction, survival, and recruitment success.

This study recorded 161 macrobenthic species in the NPP-adjacent waters, with an average abundance of 97.33 ind./m^2^ and an average biomass of 16.98 g/m^2^. These values are higher than those reported from other coastal power plant areas in Fujian and Guangdong by Cai Lizhe et al. [12], Lin Xiuchun et al. [56], and Zhang et al. [40], but lower than the higher diversity documented at the Sanmen NPP (Zhou et al. [57,58]), and the Gori NPP in Korea (Yu et al. [59]) (Table 5). The higher species number, abundance, and biomass observed in this study compared to previous surveys and adjacent sea studies may be attributed to three reasons: The 6-year survey period covered interannual cycles of macrobenthic communities, such as reproduction and migration. As emphasized by Hines and Pereira et al. [60], Magurran [61], and Menger [62], long-term monitoring data help reveal the true state of biodiversity. The higher species number compared to previous studies [12,56,63] may be related to the enhanced adaptability of heat-tolerant species induced by thermal discharge [53]. The sampling stations in this study covered areas such as the harbor basin, water intake, and drainage outlet, providing more comprehensive coverage than previous studies [12,40,56] and thus better reflecting the macrobenthic community diversity in the NPP-adjacent waters [64].

### 4.2. Potential Risk of Macrobenthos to Cooling Water System Blockage

The observed ecological shifts in the macrobenthic community have direct implications for the operational security of the Northeast Fujian NPP, specifically the risk of blockage in the cooling water system. Using the GMRI, we identified two medium-risk species for Northeast Fujian NPP cooling water blockage: *Protankyra bidentata* (spiny sea cucumber) and *Actiniaria* sp. (sea anemone). The blockage risk associated with these organisms stems from their unique morphological and behavioral traits [12,56], which determine their potential to reach and accumulate on cooling water intake screens.

*Protankyra bidentata* exhibited a persistent medium risk (GMRI 50.67–64.98%) from 2019 to 2023. This species is common in the subtidal zones of bays such as Xinghua Bay [56,65], Dayawan Bay [66], and Dagang Bay [56], and has been recognized as a dominant member of the macrobenthic community near the Northeast Fujian NPP [13,36,67] (Table 5). Its blockage risk is driven by three key biological traits. First, it lacks developed locomotive organs and possesses a soft, highly flexible body wall [68], allowing it to pass through the 5 mm screens typically used in NPP cooling systems—unlike larger, rigid-bodied Echinoderms (e.g., sea urchins). Second, its 100% floating capacity enables passive transport by currents toward the water intake, increasing the probability of screen contact. Third, its high proportional biomass (11.75–53.66% during medium-risk periods) means that even moderate population increases can lead to substantial screen accumulation.

Notably, the maximum GMRI value for *Protankyra bidentata* recorded here (64.98%) is considerably higher than the 46.34% (low risk) reported by Cai et al. [12] and Lin et al. [56]. This discrepancy may be linked to elevated nutrient levels in 2019, which promoted organic matter accumulation in surface sediments [69], thereby creating favorable habitat conditions that supported a population expansion of *Protankyra bidentata*. This finding connects nutrient-driven community changes directly to the risk of biological blockage.

In contrast, *Actiniaria* sp. presented only an episodic medium risk, with a GMRI of 54.63% recorded in July 2021. Its blockage mechanism is distinct: rather than physical accumulation, it relies on viscous secretions from its tentacles to adhere to screen surfaces [70]. These secretions form stable biofilms that directly impede water flow and can trap other small organisms (e.g., juvenile crustaceans), exacerbating blockage. The episodic nature of this risk suggests that it is triggered by short-term environmental factors, such as seasonal reproductive peaks of *Actiniaria* sp. or temporary nutrient enrichment that enhances mucus production. This pattern aligns with previous observations that the abundance of sea anemones often increases during summer and autumn [71], likely reflecting seasonal variations in key environmental drivers like temperature and nutrient availability.

### 4.3. Effects of Habitat Factors on Macrobenthic Communities

The macrobenthic community structure is fundamentally governed by a suite of abiotic habitat factors, a principle demonstrated across diverse coastal systems where factors such as salinity, nutrient loading, and sediment properties are key determinants [72]. Studies in regions like Laizhou Bay and Xiangshan Bay have shown community sensitivity to shifts in salinity and direct responses to nitrogen and phosphorus enrichment [73,74]. In estuarine systems like the Scheldt, nutrients can indirectly shape communities by altering sediment conditions [75], while near coastal infrastructure, factors including suspended solids are often primary drivers [40]. Our long-term study aligns with this established framework and identifies the specific environmental drivers operative in the NPP-adjacent ecosystem.

The Mantel test and random forest analysis identified dissolved inorganic nitrogen and phosphorus as the dominant environmental correlates of the macrobenthic community structure from 2018 to 2024. The significant positive correlation between polychaete abundance and nutrient levels provides the mechanistic basis for their persistent dominance. Elevated nutrients stimulate phytoplankton production, increasing the flux of labile organic matter to the seabed. This enrichment preferentially benefits opportunistic, deposit-feeding polychaetes such as *Notomastus* cf. *aberans* and *Heteromastus filiformis*, fueling their proliferation and numerical dominance [76,77].

Notably, nutrients (NH_4_^+^, NO_3_^−^) were a key predictor of community diversity, explaining 13.6% of the variance in the Shannon–Wiener index. This points to a process of competitive exclusion under eutrophic conditions: enhanced availability of NH_4_^+^ boosts primary production, supporting high densities of a few nutrient-tolerant polychaete species that can outcompete more sensitive taxa, thereby reducing overall community evenness and species richness [76].

This elucidated nutrient–community linkage establishes a conceptual bridge between environmental conditions and operational risk. The same nutrient dynamics that structure the overall community also indirectly modulate habitat suitability for specific, morphologically specialized blockage-risk species. For instance, periods of high nutrient loading can promote sedimentary organic matter accumulation [76], potentially creating favorable conditions for species like *Protankyra bidentata*, explaining its elevated GMRI in high-nutrient years. Therefore, monitoring key nutrients (NH_4_^+^, NO_3_^−^) serves a dual purpose: a proxy for general community state and a potential early-warning indicator for conditions that may elevate the biofouling risk posed by specific taxa.

Our study has limitations, including the potential for annual sampling to miss seasonal variations and the absence of a control site. Future work should strengthen high-frequency monitoring, develop integrated predictive models, and implement proactive management strategies based on mechanistic insights into key environmental drivers.

## 5. Conclusions

This study advances the understanding of NPP ecological impacts by demonstrating the co-dominance of thermal and nutrient drivers in shaping macrobenthic communities over a multi-year scale. The identification of *Protankyra bidentata* and *Actiniaria* sp. as medium-risk species highlights the importance of non-dominant, morphologically suited taxa in cooling system blockages. Our results provide a scientific basis for targeted monitoring of these risk species and their environmental drivers (nutrients and temperature). We recommend integrating long-term benthic community data into early-warning NPP risk systems to support proactive management of cooling water system security.

## Figures and Tables

**Table 1:** Composition of macrobenthic species from 2018 to 2024.

Species Composition	Year					
	2018	2019	2020	2021	2023	2024
Polychaetes	32	35	23	31	26	39
Crustaceans	16	12	14	15	11	12
Mollusks	5	10	10	9	4	14
Echinoderms	5	6	3	6	1	6
Other groups	6	7	6	6	3	5
Total number of species	64	70	56	67	45	76

**Table 2:** Dominant species of macrofauna in the adjacent sea area of Northeast Fujian Nuclear Power Plant (dominant species/dominance value).

Year	Species	Water Intake	Species	Harbor Basin	Species	Drain Outlet
2018	*Gammaridean* sp.	0.148	*Notomastus* cf. *aberans*	0.072	*Notomastus* cf. *aberans*	0.079
	*Xenophthalmus pinnotheroides*	0.092			*Sternaspis scutata*	0.053
	*Glycera chirori*	0.067			*Xenophthalmus pinnotheroides*	0.026
2019	*Xenophthalmus pinnotheroides*	0.097	*Protankyra bidentata*	0.185	*Notomastus* cf. *aberans*	0.125
	*Glycera chirori*	0.072	*Harmothoë* sp.	0.089	*Protankyra bidentata*	0.107
	*Lumbrineris latreilli*	0.038	*Amaeana occidentalis*	0.073	*Sternaspis scutata*	0.080
2020	*Aglaophamus dibranchis*	0.066	*Nassarius semiplicatus*	0.045	*Notomastus* cf. *aberans*	0.129
	*Paraprionospio pinnata*	0.060	*Scapharca subcrenata*	0.032	*Glycinde gurjanovae*	0.086
	*Protankyra bidentata*	0.048	*Polynoidae*	0.026	*Vibridae* sp.	0.065
2021	*Actiniaria* sp.	0.190	*Protankyra bidentata*	0.430	*Paraprionospio pinnata*	0.040
	*Caprella equilibra*	0.087	*Actiniaria* sp.	0.248	*Notomastus* cf. *aberans*	0.037
	*Nucula faba*	0.053	*Notomastus* cf. *aberans*	0.066	*Gammaridean* sp.	0.035
2023	*Heteromastus filiforms*	0.165	*Notomastus* cf. *aberans*	0.151	*Heteromastus filiforms*	0.667
	*Neoxenophthalmus obscurus*	0.072	*Sigambra bassi*	0.075	*Notomastus* cf. *aberans*	0.038
	*Sigambra bassi*	0.072	*Heteromastus filiforms*	0.071	*Sigambra bassi*	0.038
2024	*Gammaridean* sp.	0.355	*Polydora* sp.	0.694	*Sternaspis scutata*	0.082
	*Terebellides stroemii*	0.022	*Glycinde gurjanovae*	0.043	*Harmothoe imbricata*	0.070
			*Glycera chirori*	0.036	*Gammaridean* sp.	0.070

**Table 3:** Potential risk levels of macrobenthos blocking the cooling water system of Northeast Fujian NPP in adjacent waters (2018–2024).

Date	Species	A/(%)	B/(%)	P/(%)	F/(%)	C/(%)	GMRI/(%)	Risk Level
July 2018	*Notomastus* cf. *aberans*	6.45	0.87	45.83	60	90	40.63	Low Risk
July 2018	*Sternaspis scutata*	3.23	0.38	33.33	100	90	45.39	Low Risk
July 2018	*Lumbrineris latreilli*	4.52	1.14	29.17	60	90	36.96	Low Risk
July 2018	*Gammaridean*	11.61	0.18	33.33	40	90	35.03	Low Risk
July 2018	*Nephtys oligobranchia*	0.97	0.17	12.50	60	90	32.73	Low Risk
July 2018	*Actiniaria*	1.61	1.08	20.83	100	100	44.70	Low Risk
July 2018	*Protankyra bidentata*	1.61	21.42	12.50	100	100	47.11	Low Risk
July 2018	*Paraprionospio pinnata*	2.26	0.14	25.00	60	90	35.48	Low Risk
July 2018	*Terebellides stroemii*	0.32	0.05	4.17	60	90	30.91	Low Risk
July 2018	*Aglaophamus dibranchis*	2.90	0.40	25.00	60	90	35.66	Low Risk
July 2018	*Glycera chirori*	9.68	4.35	66.67	60	90	46.14	Low Risk
July 2019	*Notomastus* cf. *aberans*	6.38	2.85	54.17	60	90	42.68	Low Risk
July 2019	*Sternaspis scutata*	4.08	0.38	45.83	100	90	48.06	Low Risk
July 2019	*Lumbrineris latreilli*	3.06	0.63	20.83	60	90	34.90	Low Risk
July 2019	*Gammaridean* sp.	3.06	0.04	8.33	40	90	28.29	Extremely Low Risk
July 2019	*Nephtys oligobranchia*	1.02	0.05	12.50	60	90	32.71	Low Risk
July 2019	*Actiniaria*	1.53	0.85	25.00	100	100	45.48	Low Risk
July 2019	*Protankyra bidentata*	11.99	46.24	66.67	100	100	64.98	Medium Risk
July 2019	*Trigonothracia jinxingae*	4.85	5.07	20.83	40	30	20.15	Extremely Low Risk
July 2019	*Paraprionospio pinnata*	1.02	0.06	12.50	60	90	32.72	Low Risk
July 2019	*Terebellides stroemii*	0.26	0.03	4.17	60	90	30.89	Low Risk
July 2019	*Aglaophamus dibranchis*	2.55	1.34	25.00	60	90	35.78	Low Risk
July 2019	*Glycera chirori*	6.89	3.63	66.67	60	90	45.44	Low Risk
October 2020	*Notomastus* cf. *aberans*	5.11	0.31	17.39	60	90	34.56	Low Risk
October 2020	*Sternaspis scutata*	0.57	0.05	4.35	100	90	38.99	Low Risk
October 2020	*Gammaridean* sp.	2.84	0.05	17.39	40	90	30.06	Low Risk
October 2020	*Glycinde gurjanovae*	5.68	0.17	30.43	60	90	37.26	Low Risk
October 2020	*Actiniaria* sp.	2.84	0.47	8.70	100	100	42.40	Low Risk
October 2020	*Protankyra bidentata*	6.82	11.75	34.78	100	100	50.67	Medium Risk
October 2020	*Paraprionospio pinnata*	6.25	0.19	26.09	60	90	36.51	Low Risk
October 2020	*Terebellides stroemii*	0.57	0.13	4.35	60	90	31.01	Low Risk
October 2020	*Aglaophamus dibranchis*	7.95	0.22	34.78	60	90	38.59	Low Risk
October 2020	*Glycera chirori*	0.57	0.04	4.35	60	90	30.99	Low Risk
July 2021	*Edwardsia* sp.	0.37	0.20	8.70	100	100	41.85	Low Risk
July 2021	*Notomastus* cf. *aberans*	5.81	0.78	73.91	60	90	46.10	Low Risk
July 2021	*Sternaspis scutata*	1.50	0.67	30.43	100	90	44.52	Low Risk
July 2021	*Lumbrineris latreilli*	0.75	0.06	17.39	60	90	33.64	Low Risk
July 2021	*Gammaridean*	6.18	0.17	56.52	40	90	38.57	Low Risk
July 2021	*Glycinde gurjanovae*	1.50	0.13	17.39	60	90	33.80	Low Risk
July 2021	*Nephtys oligobranchia*	2.25	0.12	34.78	60	90	37.43	Low Risk
July 2021	*Acaudina molpadioides*	0.19	24.18	4.35	100	100	45.74	Low Risk
July 2021	*Actiniaria*	16.48	4.51	52.17	100	100	54.63	Medium Risk
July 2021	*Protankyra bidentata*	16.85	39.33	47.83	100	100	60.80	Medium Risk
July 2021	*Trigonothracia jinxingae*	1.50	2.04	30.43	40	30	20.79	Extremely Low Risk
July 2021	*Paraprionospio pinnata*	2.06	0.22	34.78	60	90	37.41	Low Risk
July 2021	*Terebellides stroemii*	0.19	0.04	4.35	60	90	30.91	Low Risk
July 2021	*Aglaophamus dibranchis*	1.50	0.16	21.74	60	90	34.68	Low Risk
July 2021	*Glycera chirori*	0.56	0.04	13.04	60	90	32.73	Low Risk
October 2023	*Notomastus* cf. *aberans*	9.66	1.39	8.70	60	90	33.95	Low Risk
October 2023	*Sternaspis scutata*	1.49	1.83	43.48	100	90	47.36	Low Risk
October 2023	*Listrolobus brevirostris*	0.59	2.84	13.04	100	100	43.30	Low Risk
October 2023	*Lumbrineris latreilli*	1.63	0.28	13.04	60	90	32.99	Low Risk
October 2023	*Gammaridean* sp.	4.46	0.26	8.70	60	90	32.68	Low Risk
October 2023	*Glycinde gurjanovae*	4.09	0.36	17.39	60	90	34.37	Low Risk
October 2023	*Nephtys oligobranchia*	4.53	0.35	4.35	60	90	31.85	Low Risk
October 2023	*Protankyra bidentata*	4.53	53.66	4.35	100	100	52.51	Medium Risk
October 2023	*Paraprionospio pinnata*	0.30	0.04	4.35	60	90	30.94	Low Risk
October 2023	*Glycera chirori*	0.45	0.17	30.43	60	90	36.21	Low Risk
July 2024	*Notomastus* cf. *aberans*	2.50	0.44	34.78	60	90	37.55	Low Risk
July 2024	*Sternaspis scutata*	3.68	1.19	34.78	100	90	45.93	Low Risk
July 2024	*Polydora* sp.	17.89	0.75	43.48	60	90	42.42	Low Risk
July 2024	*Lumbrineris latreilli*	0.79	0.13	17.39	60	90	33.66	Low Risk
July 2024	*Gammaridean*	28.68	0.69	47.83	40	90	41.44	Low Risk
July 2024	*Glycinde gurjanovae*	2.24	0.25	39.13	60	90	38.32	Low Risk
July 2024	*Nephtys oligobranchia*	2.24	0.25	43.48	60	90	39.19	Low Risk
July 2024	*Protankyra bidentata*	2.89	18.62	17.39	100	100	47.78	Low Risk
July 2024	*Trigonothracia jinxingae*	1.84	3.85	17.39	40	30	18.62	Extremely Low Risk
July 2024	*Paraprionospio pinnata*	0.13	0.15	4.35	60	90	30.93	Low Risk
July 2024	*Terebellides stroemii*	2.50	1.81	26.09	60	90	36.08	Low Risk
July 2024	*Aglaophamus dibranchis*	0.66	0.10	17.39	60	90	33.63	Low Risk
July 2024	*Glycera chirori*	3.42	2.56	52.17	60	90	41.63	Low Risk

**Table 4:** Water temperature at water intake, drain outlet and harbor basin from 2018 to 2024.

Year	Water Intake/(°C)	Harbor Basin/(°C)	Drain Outlet/(°C)
2018	30.9 ± 0.1	30.1 ± 0.2	33.3 ± 1.3
2019	30.5 ± 0.1	29.9 ± 0.1	30.7 ± 0.6
2020	23.7 ± 0.1	23.7 ± 0.1	27.6 ± 1.5
2021	30.1 ± 0.2	30.0 ± 0.1	33.7 ± 1.2
2023	28.3 ± 0.1	27.6 ± 0.1	29.7 ± 1.3
2024	24.1 ± 0.1	23.8 ± 0.6	26.0 ± 1.1

**Table 5:** Species richness, abundance, and biomass of macrobenthic communities in adjacent waters of different nuclear power plants.

Study Area	Number of Stations	Survey Period	Total Number of Species	Number of Species in the Top Three Taxa	Average Density (ind./m^2^)	Average Biomass (g/m^2^)	Main Potential Risk Organisms	GMRI	References
Qingchuan Bay Waters, Fujian	24	July 2018–July 2024	161	Polychaetes 71 species	97.33	16.98	*Protankyra bidentata*	47.11–64.98%	This Study
				Crustaceans 35 species					
				Mollusks 30 species					
Xinghua Bay Waters, Fujian	16	June 2019	85	Polychaetes 54 species	/	/	*Phyllophorus gangeticus*	63.78%	Lin X.C. [56]
				Crustaceans 15 species					
				Mollusks 11 species					
Qingchuan Bay Waters, Fujian	24	January–April, July, October 2018	54–69	Polychaetes 32 species	37.50–77.50	7.44–11.07	*Protankyra bidentata*	47.11%	Zhang W.B. [40]
				Crustaceans 16 species					
				Mollusks 5 species					
Dayawan Bay, Guangdong	16	March 2021	102	Polychaetes 45 species	/	/	*Timoclea scabra*	65.52%	Cai L.Z. [12]
				Mollusks 30 species			*Listriolobus brevirostris*	51.88%	
				Crustaceans 10 species					
Changjiang Nuclear Power Plant, Hainan	15	December 2018	67	Polychaetes 35 species	/	/	*Aglaophamus dibranchis*	50.86%	Lin X.C. [56]
				Mollusks 11 species					
				Crustaceans 10 species					
Sanmen Nuclear Power Plant, Zhejiang Province, China	12	Autumn 2022, Spring 2023, Summer 2023, Winter 2024	43	Polychaetes 21 species, Arthropoda 8 species, Echinodermata 4 species	300–800	50–150	*Nassarius succinctus* *Nassarius variciferus*	/	Zhou W.W. [57,58]
Gori Nuclear Power Plant, Korea	16	September, October 2011, February, April, May 2012	369	Polychaetes 155 species Mollusks 82 species Crustaceans 82 species	1712	135	*Magelona japonica*	/	Yu, O.H. [59]

All samples were collected from a 0.2 m^2^ area using a 0.5 mm mesh.

## Data Availability

The original contributions presented in this study are included in the article. Further inquiries can be directed to the corresponding authors.
